# Notes and News

**Published:** 1891-11-21

**Authors:** 


### NOTES AND NEWS.

COLLECTED AND COMMUNICATED.

MR. CONRAD W. THIES (secretary of the Royal Free Hospital) will read a paper on "A Model Hospital—the New General Hospital, Hamburgh," before The Hospitals Association on Wednesday, the 25th inst., at the Royal Free Hospital, Gray's Inn Road, when Mr. Jonathan Hutchinson will take the chair at 8 p.m.

AT the Pathological Society of London, on the 17th inst., Dr. Walter Sibley read a paper on "Inoculated Tuberculosis in Snakes." He stated that after failures he had at last succeeded in producing tuberculosis in snakes by inoculating them with tubercle bacilli, and then keeping them in a warm atmosphere. He described two cases of the disease produced in this way; in the one case the snake died fifty-two, and in the other seventy-two days after the primary inoculation. In both cases the deposits were most abundant in the liver, and all of the lesions were crowded with baccilli. He referred to a case of spontaneous tuberculosis in a snake from the Zoological Society's Gardens, a description of which he had published in Virchow's Archives, and concluded his paper by saying that tuberculosis could no longer be considered a disease confined to the higher or warm-blooded animals, but that the same germ might produce the same disease in snakes as in the human subject. In fact, he saw no reason why consumption could not be artificially produced in some of the invertebrate forms of animal life, and he had already carried on some experiments in this direction, the results of which he hoped soon to publish.

THE Greenwich District Board of Works, in view of the serious outbreak of typhoid fever in Deptford and Greenwich, has submitted samples of the water supplied to the district by the Kent and Southwark and Vauxhall Water Companies for Analysis by Dr. Stephenson and Dr. Washbourne. The water was taken from those parts of the districts which were particularly affected by the epidemic. The analysts come to the conclusion that the water from the Southwark and Vauxhall Company's supply was impure and of undesirable quality for public consumption. There was no evidence, however, of the presence of matter of a specifically injurious character. The water supplied by the Kent Company, except in one case, where it was evident the cistern was dirty, was pure. There is good reason to believe that the epidemic is rapidly on the decline, for, although no fewer than 202 cases have been reported in Deptford alone during the past four weeks, the greater number occurred in the first fortnight, and only 52 cases in the last two weeks. In Greenwich only seven cases of typhoid have been reported to the Medical Officer in the past month. Forty-two cases of scarlet fever and 10 of diphtheria have been reported in Deptford within the past four weeks.

THE Isolation Hospital Scheme for Enfield has developed a very acute stage during the past week, and the alarm and anxiety which the Local Board have excited in the minds of a large section of the Enfield public is not likely to be lessened by the indignation meeting held there last week. When everybody were thinking that, so far as Enfield was concerned, a satisfactory solution had at length been reached as to the vexed question of the locale for the hospital, and anticipations of a speedy acquisition of a too long delayed *desideratum* were dormant, at a great cost to the ratepayers, the Enfield Local Board suddenly determined to acquire a piece of land on the borders of Bush Hill Park for the erection of a temporary fever hospital. This determination was arrived at without public discussion, and without, apparently, the knowledge of several members of the Board itself. Other sites have been proposed, and objections to them considered. In this case, however, the recommendation of the medical officer and, we suppose, of other officials of the Enfield Local Board, has been deemed sufficient. Considerations of local conditions and feeling, and of general expediency have not been allowed to enter into the question at all. The spot chosen is stated to be in a swampy hollow, with an environment of mud, and approached by dirty lanes. It is true the Board proposes spending money upon the making up of the approaches. It is, however, questionable whether moneys should thus be spent for a temporary purpose. It is also questionable whether a populous and poor locality is a suitable one for such an institution. Nine hundred pounds is a large sum to expend on a doubtful and temporary expedient, and it is to be hoped that some more satisfactory arrangement will be arrived at.

THE report of Dr. W. Collingridge, the Medical Officer of Health for the Port of London, for the half-year ending June 30th, 1891, has just been published, and gives some interesting information with reference to the work of sanitation as carried out in the Port of London. The total number of vessels inspected by the Port Sanitary staff during the six months was 7,608. Legislation does not provide that designs of new vessels should be submitted to sanitary inspection, and owners are frequently put to expense for subsequent alterations. Small-pox has been reported on three vessels only. This absence of small-pox has rendered it possible to trace individual cases, one instance illustrating with tolerable clearness that the contagion may be carried through the air for a distance of at least a quarter of a mile. The sanitary arrangements necessary on the housing, during the strike in the winter months, of a large number of labourers within the the docks, caused the Port Sanitary Authority much anxiety. It was felt to be most important to remain absolutely neutral in the question, insisting upon the requirements necessary for public health; not in any way to place prohibitive restrictions in the way of the employers. The particulars as to how they were carried out are given in detail and are most interesting, as also is the very exhaustive account which Dr. Collingridge gives of the Atlantic cattle ships. The question of the river water is again to the fore. The Medical Officer gives it as his opinion that the London County Council should be given time to perfect and test the works now in course of construction. The operations already carried out have in no wise improved the condition of the river water, which remains as heretofore, entirely dependent on climatic influence. He, however, is by no means sanguine as to the result which will follow the completion of the work, which has been so freely condemned by most sanitary experts.

